# Supplementary material for: MetaRibo-Seq measures translation in microbiomes
Source: Nat Commun. 2020 Jun 29;11:3268. doi: 10.1038/s41467-020-17081-z (PMC7324362; doi:10.1038/s41467-020-17081-z)
Supplement: Supplementary file 10 — Supplementary Data 7 [file 41467_2020_17081_MOESM10_ESM.zip › File2/Confidence_VeryHigh_Taxonomy/291373_out.krona.html]

Javascript must be enabled to view this page.

members
magnitude
magnitudeUnassigned
count
unassigned
taxon
rank

291373\_out

24

24
2
superkingdom

phylum
1224
24

1236
class
24

24
order
91347

543
family
24

20
genus
561

562
species
20

SRS012273\_contig\_number\_44737SRS017191\_contig\_number\_contig-100\_6267.99740SRS017521\_contig\_number\_42154SRS019068\_contig\_number\_contig-100\_2302.487792SRS019129\_contig\_number\_4865SRS019601\_contig\_number\_15138SRS046502\_contig\_number\_contig-100\_736.737SRS049402\_contig\_number\_16673SRS064276\_contig\_number\_2582SRS075947\_contig\_number\_11910SRS076876\_contig\_number\_contig-100\_1099.1100SRS077454\_contig\_number\_contig-100\_463.92013SRS098881\_contig\_number\_14974SRS140492\_contig\_number\_30568SRS142781\_contig\_number\_6828SRS143014\_contig\_number\_contig-100\_523.524SRS144362\_contig\_number\_17647SRS146888\_contig\_number\_13784SRS147022\_contig\_number\_14704SRS147151\_contig\_number\_contig-100\_6749.102549

1
genus
547

1

SRS1055050\_contig\_number\_contig-100\_518.519
species
539813

1

SRS046717\_contig\_number\_contig-100\_16221.60911
genus
3
570

573
species
2

SRS077646\_contig\_number\_contig-100\_2747.2748SRS143070\_contig\_number\_contig-100\_909.75681
